# Acute Panic Attack During Spinal Anesthesia: Successful Management With Intraoperative Psychological Intervention

**DOI:** 10.7759/cureus.111343

**Published:** 2026-06-23

**Authors:** Joshua Nmezi

**Affiliations:** 1 Anesthesia, Pilgrim Hospital, Boston, GBR; 2 Anesthesia and Clinical Psychology, Imo State University, Owerri, NGA

**Keywords:** case report, intraoperative anxiety, panic attack, psychological intervention, regional anaesthesia, spinal anaesthesia

## Abstract

Spinal anesthesia is widely used for lower abdominal and orthopedic procedures, offering advantages including active patient cooperation and reduced systemic effects. However, some patients experience significant anxiety during awake regional anesthesia, and a minority may develop acute panic attacks. We report the case of a 52-year-old woman with no prior psychiatric history who underwent elective total knee replacement under spinal anesthesia. Approximately 35 minutes after successful spinal anesthesia, she developed acute symptoms including severe anxiety, palpitations, chest tightness, dyspnea, a feeling of impending doom, and uncontrollable trembling. Vital signs revealed tachycardia (142 bpm) and hypertension (180/95 mmHg), while oxygen saturation remained within normal limits. The surgical team considered aborting the procedure and converting to general anesthesia. Immediate bedside psychological intervention was provided by the attending anesthetist (with clinical psychology training), consisting of grounding techniques, breathing retraining, cognitive reassurance, and guided imagery. Symptoms resolved within eight minutes, and surgery proceeded uneventfully. This case highlights the importance of recognizing panic attacks in the intraoperative setting and demonstrates that brief psychological intervention can avert procedure abandonment and facilitate successful completion of surgery.

## Introduction

Spinal anesthesia is a safe and effective technique for lower limb and lower abdominal surgery, with advantages including avoidance of airway manipulation, reduced postoperative nausea, and early mobilization [1]. However, the requirement for patients to remain awake during surgery can be anxiety-provoking, and a notable subset of patients may experience significant distress [2]. Several factors contribute to this anxiety, including the loss of control over the lower body, the unfamiliar sensation of numbness, and the exposure to surgical sights, sounds, and smells while fully conscious. These elements can create a sense of vulnerability that predisposes susceptible individuals to acute psychological distress.

The prevalence of anxiety during regional anesthesia is estimated at 10-20%, with a smaller subset experiencing acute panic attacks [3]. A panic attack is defined as an abrupt surge of intense fear or discomfort that peaks within minutes, accompanied by physical and cognitive symptoms including palpitations, sweating, trembling, shortness of breath, chest pain, nausea, dizziness, and fear of losing control or dying [4].

When panic occurs intraoperatively, it poses immediate challenges. Patients may become uncooperative, vital signs may become unstable, and the surgical team may feel compelled to prematurely convert to general anesthesia to ensure patient safety [5]. Standard anesthetic responses such as intravenous sedation or conversion to general anesthesia may be effective but carry additional risks, including respiratory depression, airway management challenges in the middle of surgery, prolonged recovery, and the potential for hemodynamic instability during induction. Furthermore, these approaches do not address the underlying psychological distress, leaving the root cause of the episode unmanaged.

We report a case of an acute panic attack during spinal anesthesia that was successfully managed with a brief, targeted psychological intervention, allowing surgery to proceed without conversion to general anesthesia.

## Case presentation

Patient information

A 52-year-old woman presented for elective right total knee replacement for severe osteoarthritis. She had no prior psychiatric history, including no previous panic attacks or anxiety disorders. Past medical history included hypertension managed with amlodipine 5 mg daily. She had no previous surgical history.

Preoperative assessment

Preoperative assessment was unremarkable (Table 1). The patient appeared calm and reported no anxiety about the procedure. She specifically requested spinal anesthesia based on a friend's recommendation. No preoperative psychological screening was conducted.

**Table 1:** Preoperative laboratory findings.

Parameters	Result	Reference range
Hemoglobin	13.2 g/dL	12.0-16.0 g/dL
White blood cell count	6.8 x 10⁹/L	4.0-11.0 x 10⁹/L
Platelets	245 x 10⁹/L	150-450 x 10⁹/L
Sodium	138 mmol/L	135-145 mmol/L
Potassium	4.1 mmol/L	3.5-5.0 mmol/L
Urea	5.2 mmol/L	2.5-7.5 mmol/L
Creatinine	82 µmol/L	60-110 µmol/L
Glucose (random)	5.4 mmol/L	<7.8 mmol/L
Calcium	2.3 mmol/L	2.2-2.6 mmol/L
Magnesium	0.85 mmol/L	0.7-1.0 mmol/L

Initial intraoperative course

Spinal anesthesia was performed at the L3-L4 interspace using 2.5 mL of 0.5% heavy bupivacaine with 25 mcg fentanyl. Sensory block to T10 was achieved. The patient was positioned supine, and surgery commenced. Initial vital signs were stable (heart rate: 78 bpm, blood pressure: 128/74 mmHg).

Approximately 35 minutes after spinal injection, the patient suddenly became agitated. She reported an inability to breathe, a sense that something terrible was happening, and a feeling that she was going to die, accompanied by chest tightness and palpitations. Observed signs included trembling of the upper extremities, diaphoresis, rapid shallow breathing, and an inability to follow instructions. 

Vital signs revealed tachycardia (142 bpm), hypertension (180/95 mmHg), and tachypnea (28 breaths/min). The sensory level of the block at this time was assessed as T8. Oxygen saturation remained normal at 98% on room air, and electrocardiography showed sinus tachycardia without ectopy. Pupillary examination revealed bilateral midsized pupils that were sluggish to react, consistent with sympathetic activation rather than neurological compromise. The patient's skin was flushed and warm to the touch, which further supported a diagnosis of panic-related sympathetic surge rather than anaphylaxis or high spinal block.

The surgical field was dry, and there was no evidence of high spinal (the highest level of block was T8), local anesthetic toxicity, or hemorrhage.

**Figure 1:**
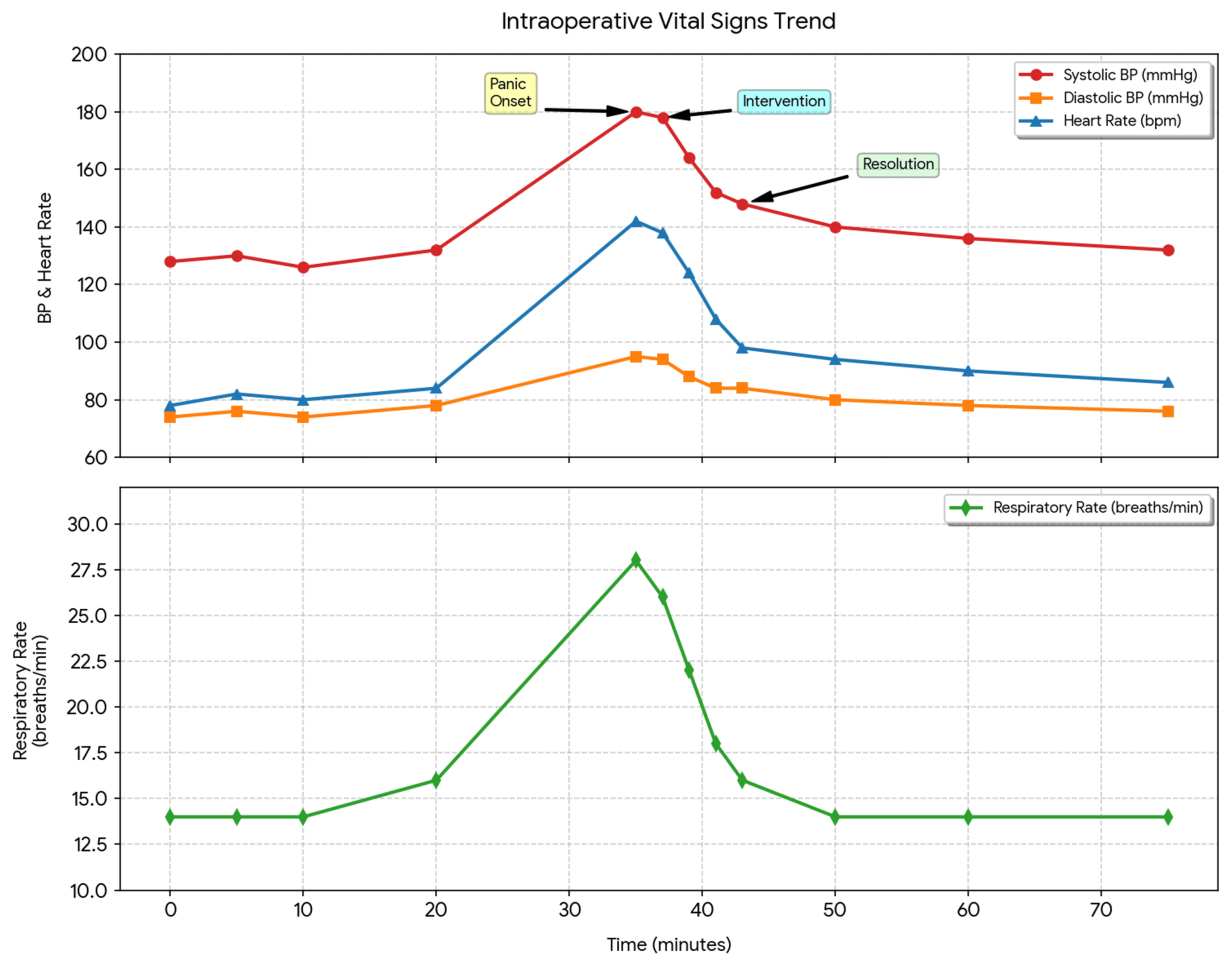
Intraoperative vital signs trend.

A comprehensive differential diagnosis was considered. High spinal cord involvement was ruled out by stable blood pressure and preserved upper-limb motor function. Local anesthetic toxicity was unlikely given the absence of prodromal symptoms or seizures and the timing of presentation. Anaphylaxis was excluded due to the lack of rash, bronchospasm, or hypotension. Pulmonary embolism was considered but deemed unlikely given the absence of hypoxia, acute onset, and lack of risk factors. The clinical presentation was most consistent with an acute panic attack. Intraoperative vital signs trend is shown in Figure 1.

Psychological intervention

Recognizing the presentation as consistent with an acute panic attack, the attending anesthetist (author JN, with clinical psychology training) initiated immediate psychological intervention while continuing to monitor vital signs:

Step 1 - Grounding (approximately two minutes): The clinician used a calm, reassuring tone, stating, "You are safe. This is a panic attack. It will pass." The patient was directed to look around the room and name objects she could see, and a cool cloth was placed on her forehead as a sensory anchor to help redirect her attention from internal sensations to the external environment.

Step 2 - Breathing retraining (approximately two minutes): The patient was instructed to breathe in for a count of four, hold for four, and breathe out for four. The clinician demonstrated the technique and breathed alongside her, which helped gradually slow her respiratory rate from 28 to 16 breaths per minute.

Step 3 - Cognitive reassurance (approximately two minutes): The clinician normalized the experience by explaining that this reaction happens to some people during spinal anesthesia. The physical sensations, including tachycardia and trembling, were explained as effects of adrenaline rather than signs of danger. Finally, the patient was reassured that surgery could continue once she felt ready to proceed.

Step 4 - Guided imagery (approximately two minutes): The patient was directed to imagine a safe, calm place, specifically her garden, and was encouraged to describe the sensory details she experienced there, including the sights, sounds, and smells.

Within eight minutes, the patient's heart rate decreased to 98 beats per minute, and her blood pressure improved to 148/84 mmHg. The trembling ceased completely, and she reported feeling "calm enough to continue" with the procedure.

The remaining procedure was completed uneventfully, with a total operative time of 75 minutes. The patient remained calm throughout the remainder of the case, and no additional sedation was required.

Postoperative course

The patient was monitored in recovery for two hours with stable vital signs. She had a full recall of the panic episode. She was discharged on postoperative day three with written information about panic attacks and advice to discuss the episode with her general practitioner; an offer of psychological follow-up was declined. At four-week follow-up, she reported no further panic symptoms and was satisfied with her surgical outcome. A further telephone review was conducted at six months, at which point the patient remained well and reported no recurrence of panic symptoms in any setting, including subsequent healthcare encounters.

## Discussion

This case illustrates that acute panic attacks can occur during spinal anesthesia even in patients with no prior psychiatric history, and that brief psychological intervention can be effective in the intraoperative setting. While anxiety during regional anesthesia is common, affecting an estimated 10-20% of patients [3], acute panic attacks are less frequently reported and may be under-recognized, with symptoms often attributed to physiological causes or dismissed as "nerves" [5]. The abrupt onset, intensity, and constellation of symptoms observed in this case met Diagnostic and Statistical Manual of Mental Disorders (DSM-5) criteria for panic attack [4].

Several factors likely contributed to the panic episode. These included the loss of control associated with being awake but unable to move the lower body, sensory dissociation from unfamiliar sensations of numbness, and the operative environment with its surgical sights, sounds, and smells. A catecholamine surge from the acute stress response probably amplified the physical symptoms experienced [6]. The absence of prior psychiatric history suggests this was a situational panic attack rather than a manifestation of an underlying panic disorder.

Differentiating a panic attack from serious physiological emergencies in the intraoperative setting is critical [7]. Key distinguishing features in this case included the absence of hypoxia, hypotension, or electrocardiographic changes, together with normal oxygen saturation throughout the episode. Pupillary examination revealed bilateral midsized pupils with sluggish reactivity, consistent with sympathetic activation rather than the pinpoint pupils that might suggest opioid toxicity or the fixed dilated pupils that could indicate neurological compromise. The patient's skin was flushed and warm to the touch, which further differentiated the presentation from anaphylaxis (which typically presents with rash, bronchospasm, and hypotension) or high spinal block (which would present with hypotension, bradycardia, and progressively ascending sensory loss). The rapid resolution with psychological intervention and the patient's report of fear and sense of doom further supported the diagnosis of panic attack rather than an organic emergency.

This case demonstrates that psychological techniques can be adapted for intraoperative use. Key elements included grounding, which involves redirecting attention from internal sensations to the external environment [8]; breathing retraining to break the hyperventilation cycle [9]; cognitive reassurance to normalize and explain physical sensations [10]; and guided imagery to engage calming mental imagery [11]. While these techniques were delivered by a clinician with dual training in anesthesia and clinical psychology, basic grounding and breathing interventions can potentially be used by any anesthetist. A simple framework for clinicians without formal psychology training is the "3-step PACER" approach: Pause (stop and assess the situation), Acknowledge (name the distress and reassure the patient), Calm (initiate slow breathing), Engage (direct attention externally), and Reassure (normalize the experience and explain physical sensations) [12]. This framework can be learned in a brief training session and applied at the bedside without specialized equipment.

Few case reports describe intraoperative panic management, as most literature addresses preoperative anxiety or postoperative psychological complications [12]. A small number of similar cases have been reported, including a case of panic attack during spinal anesthesia for cesarean section managed with verbal reassurance and breathing techniques, and a case of intraoperative panic during awake craniotomy that was successfully managed with cognitive-behavioral techniques [13,14]. These reports, together with the present case, suggest that psychological first aid can be effective across different surgical contexts and should be considered as a first-line response to intraoperative distress.

Standard anesthetic responses, such as intravenous sedation or conversion to general anesthesia, could have been effective and remain appropriate options in certain circumstances. However, these approaches carry risks that should be carefully weighed [15]. Midazolam or propofol sedation could prolong recovery and obscure neurological assessment, while conversion to general anesthesia in the middle of surgery introduces risks of airway management difficulties, aspiration, hemodynamic instability during induction, and the need for additional monitoring and equipment. The psychological intervention employed in this case avoided these drawbacks while directly addressing the underlying distress. We therefore recommend that psychological first aid be considered as an initial, low-risk intervention before proceeding to pharmacological or anesthetic rescue, though we acknowledge that sedation or conversion may still be necessary in cases of severe agitation or when psychological techniques are ineffective.

This report has several limitations. As a single case, findings cannot be generalized to the broader population of patients undergoing regional anesthesia. The intervention was delivered by a clinician with specialized training in both anesthesia and clinical psychology, which may not be widely available, and the patient's long-term psychological outcome was only followed up to six months. Additionally, the patient declined formal psychological follow-up, limiting our ability to assess whether this episode was an isolated event or a precursor to future anxiety.

Several implications for clinical practice arise from this case. Anesthetists should be aware that panic attacks can occur during regional anesthesia and should maintain a high index of suspicion when patients present with acute distress. Basic psychological first aid skills, including grounding techniques, breathing retraining, and cognitive reassurance, should be incorporated into anesthesia training to equip clinicians with tools to manage such presentations. A simple mnemonic such as the "3-step PACER" framework described above can facilitate rapid recall and application in stressful situations. Multidisciplinary collaboration involving anesthesia, psychology, and nursing can enhance patient care by integrating psychological expertise into the perioperative setting. Finally, conversion to general anesthesia should not be the automatic response to intraoperative distress, as brief psychological intervention may offer a less invasive alternative that addresses the underlying cause while allowing surgery to proceed.

Prospective studies are needed to examine the incidence of intraoperative panic and the effectiveness of brief interventions. Training programs for anesthetists in basic psychological techniques should be developed and evaluated to improve patient care and outcomes in this area.

## Conclusions

This case demonstrates that acute panic attacks can occur during spinal anesthesia even in patients without prior psychiatric history. Brief, targeted psychological intervention delivered in the operating theater can effectively resolve symptoms and allow surgery to proceed without conversion to general anesthesia. Anesthetists should be equipped with basic psychological first aid skills to manage such presentations, and conversion to general anesthesia should not be the automatic response to an anxious or agitated patient during regional anesthesia.
